# Laser Surface Alloying of Sintered Stainless Steel

**DOI:** 10.3390/ma15176061

**Published:** 2022-09-01

**Authors:** Agata Dudek, Barbara Lisiecka, Norbert Radek, Łukasz J. Orman, Jacek Pietraszek

**Affiliations:** 1 Department of Material Engineering, Faculty of Production Engineering and Materials Technology, Czestochowa University of Technology, Al. Armii Krajowej 19, 42-200 Czestochowa, Poland; 2 Faculty of Mechatronics and Mechanical Engineering, Kielce University of Technology, Al. Tysiąclecia Państwa Polskiego 7, 25-314 Kielce, Poland; 3 Faculty of Environmental, Geomatic and Energy Engineering, Kielce University of Technology, Al. Tysiąclecia Państwa Polskiego 7, 25-314 Kielce, Poland; 4 Faculty of Mechanical Engineering, Cracow University of Technology, Al. Jana Pawła II 37, 31-864 Cracow, Poland

**Keywords:** sintered stainless steel (SSS), Cr_3_C_2_–NiCr coating, APS method, laser alloying, surface geometric structure (SGS), microhardness

## Abstract

A characteristic feature of sintered stainless steel (SSS) is its porosity. Porosity results in a lower density of steel, making attractive components for producing lightweight structures and materials used in industry (e.g., the automotive industry or aerospace). Scientists also observe that porosity adversely affects steel’s properties, especially its strength properties. One of the proposals for improving the discussed properties is the use of surface treatment of sintered stainless steels, e.g., with the use of concentrated energy sources such as plasma beams or laser beams. However, this proposal is an incidental subject of research, which is not justified from the point of view of the obtained research results presented by a few research groups. In this study, the surface modification (surface treatment) of sintered stainless steel was presented. The authors proposed the use of two surface treatments in order to compare them and obtain the best results. The first treatment was the deposit of Cr_3_C_2_–NiCr coatings on SSS surfaces using the atmospheric plasma spraying (APS) method. The second treatment was to create surface layers on SSSs by laser alloying the surface with a CO_2_ laser. Due to high precision and ease of automation, the most common methods in surface alloying treatment are laser technologies. This research’s main aim was to analyze the microstructure and strength properties of the SSS surface layer. The research confirms that applying the Cr_3_C_2_–NiCr coating and modifying the surface layer through the laser alloying method improves the mechanical properties of SSSs.

## 1. Introduction

Currently, the most dynamically developing group of engineering materials are sintered stainless steels produced using powder metallurgy technology (PM technology) [1,2,3,4]. The possibility of obtaining a two-phase structure with different proportions of austenite and ferrite, being the two basic structural components, is a valuable advantage of sintered stainless steels [5,6,7]. Lailatul P. H. and Maleque M. A. subjected [8] duplex stainless steel to an appropriate modification to make this material suitable for tribological and high-temperature applications. For this purpose, surface modification using fine SiC powder preplacement by the TIG torch technique has been performed. In turn, we showed in [9,10,11] the advantages of modifying sintered duplex steels by alloying the surface of the steel with Cr_3_C_2_ powder. Oke S. et al. reviewed [12] the impact of spark plasma sintering process parameters on microstructure, phase evolution, and mechanical properties of reinforced stainless steels. Attention was paid to the influence of alloying elements and ceramic reinforcements, their dispersion into the stainless steel matrix, and the importance of the matrix-reinforcement interface. A combination of properties (e.g., very good mechanical strength or corrosion resistance) makes SSS enjoy more and more interest and causes it to be a very attractive material for numerous applications [13,14,15,16]. For example, Kovaci H. and Seçer Y., in their research [17], proved the improvement of the service life of AISI 316L biomedical implants by nitriding followed by polishing and Ti-C:H sputtering. The paper [18] presents the improvement of mechanical properties of 316L stainless steel (e.g., including strength and plasticity), and thus more possibilities for industrial application, through the selection of appropriate remelting process parameters. The main problem limiting the applicability of sintered stainless steels is, among other things, their porosity [19,20,21,22]. Considering the continuous development of surface treatment technology, the surface of sintered stainless steel can be modified to improve its functional properties [23,24,25,26,27,28]. Surface treatment processes using concentrated energy sources, including lasers [29,30,31,32,33,34], are one of the most frequently used techniques for enhancing surface properties in the case of SSS [35,36,37,38,39,40,41].

Brytan Z. et al. [42] conducted studies to investigate the effect of laser surface alloying with SiC powder on microstructural changes and properties of vacuum sintered austenitic X2CrNiMo17-12-2, ferritic X6Cr13 and duplex X2CrNiMo22-8-2 stainless steels. The obtained test results showed that the surface quality of SiC was satisfactory for ferritic and austenitic stainless steels after laser alloying, but in the case of duplex stainless steel and low laser beam powers, the superficial porosities were drastically higher.

In turn, in a study of sintered austenitic stainless steel (316L), Brytan Z. and Pakieła W. [43] decided to melt its surface with a laser with Inconel 625 powder using a fibre optic laser. The process parameters were selected to melt and fully dissolve the alloying powder into the alloyed surface. The test results showed the elimination of the porosity of SSS, and a uniform distribution of nickel and molybdenum in the entire alloyed zone was obtained. In addition, an increase in superficial hardness, microhardness and surface wear resistance was confirmed.

Oke S. R. et al. [13] used laser surface alloying with boron, and some metallic elements were used in order to form the surface layers of austenitic 316L steel with improved wear behaviour. The use of the mixtures of boron and selected metallic elements as the alloying materials has been shown to reduce the laser beam power in order to obtain layers of acceptable quality, i.e., devoid of defects typical of laser processing (microcracks, gas pores). Moreover, the thickness of laser-alloyed layers (308–432 μm) was significantly higher than that produced using diffusion boriding techniques.

Du J. et al. [26] studied the influences of plasma arc remelting on the microstructure and properties of thermal sprayed Cr_3_C_2_-NiCr/NiCrAl composite coating. Based on the obtained results, it was found that the coating adhesion, microhardness, and wear resistance were improved by plasma arc remelting with limited power (below 42 kW). The adhesion has been improved due to the enhancement of the element diffusion near the interface and metallurgical bonding between the functional and bonding layers. In addition, the increase in microhardness is attributed to the decrease in porosity and the formation of secondary carbides and the eta phase.

Schwanekamp T. et al. [28] considered qualifying chromium carbide (Cr_3_C_2_–NiCr), which is widely used for coating applications and commonly processed through thermal spraying, for use in laser beam melting (LBM) process technology. It has been shown that pre-heating can significantly improve the material quality. In addition, it has been shown to reduce porosity and prevent thermal cracking with low laser energy input. Measurements of the microhardness of the laser molten material indicated a hardness close to the specifications of thermally sprayed material.

It should be emphasized that the surface treatment (surface alloying) of sintered stainless steels is an incidental subject of research. Only a few works deal with this subject, mainly based on the use of the laser as a concentrated source of energy to constitute the properties of the surface layer of sintered steels.

The authors of this paper propose a new approach, which has not been used so far, consisting of the use of two surface treatments (APS and LSA methods) of SSS steel with the use of a ceramic material (Cr_3_C_2_ carbide). On the basis of the obtained test results, it was found that the created coatings and layers had a positive effect on the improvement of the mechanical properties of sintered stainless steels. The geometric structure of the resulting modified surface was subjected to a detailed analysis because they are considered to largely influence several phenomena that take place within the analyzed layer [44,45,46].

## 2. Materials and Methods

### 2.1. Materials

The water-atomized powders were used to obtain the multiphase stainless sinters. The powders were manufactured by Höganäs (Höganäs Municipality, Sweden) from AISI 316L steel and AISI 409L steel. The chemical composition of the steel powders is shown in Table 1. For both types of powder, the nominal particle size was 150 µm.

In the investigation, the powders were mixed in three different proportions of ferritic and austenitic steel powders. Details of the proportion are presented in Table 2.

The sintering of samples was carried out at the temperature of 1250 °C for 30 min. Then they were cooled down at a cooling rate of 0.5 °C/s. The powders used were compacted uniaxially with the addition of 1% Acrawax C lubricant at 720 MPa. The entire process was carried out in a reducing atmosphere with the use of 100% hydrogen. This approach significantly reduces the oxidation of the batch and prevents an undesirable reduction in chromium content.

### 2.2. Surface Treatment Techniques

In order to improve the functional properties, the surfaces of multiphase sintered stainless steels were treated with the use of concentrated energy sources: plasma and laser. Plasma spraying (APS) was used to apply a coating modifying the surface layer. Chromium carbide, previously mechanically mixed in a proportion of 75% wt. Cr_3_C_2_ and 25% NiCr, was selected as the coating material. This coating was prepared at the following parameters:

Voltage: Ur = 50 V,

Current intensity: Ir = 45 A,

Distance of the plasmatron from the surface: 120 mm,

Plasma forming gases flow: argon ~45 L/min and hydrogen ~12 L/min.

The resulting coating had a thickness of around 60 µm.

The laser alloying (LSA) of sintered stainless steel was carried out at the Laser Center of the Kielce University of Technology Laboratory. To produce surface layers, pulsed laser alloying was carried out by a TRUMPF Lasercell 1005 (Ditzingen, Germany). The CO_2_ laser generator has a power of 3800 W. The stand has a modern gas installation with a mixer for shielding gases. The control system is based on a Siemens Sinumerik 840D postprocessor (München, Germany). Table 3 presents the parameters chosen experimentally for the laser alloying process.

The laser alloying parameters were established in accordance with the guidelines of the manufacturer of the laser device and the previous experience of the authors. The laser treatment was performed in an atmosphere of argon as a protective gas. The tests used a focusing head. The TEM_00_ beam defined the radiation energy distribution.

### 2.3. Macro Nad Microstructure Analysis

After applying Cr_3_C_2_–NiCr powder and the surface alloying process, sintered stainless steels were subjected to macroscopic evaluation using the Olympus SZ61 stereomicroscope (Shinjuku, Japan). The microstructure was analyzed using the light optical microscope Olympus GX41 and scanning electron microscope Jeol JSM–6610LV (Akishima, Japan).

### 2.4. Chemical and Phase Composition Analysis

The chemical composition analysis was carried out by scanning electron microscope Jeol JSM–6610LV.

To determine the phase composition of the alloyed surface of sintered stainless steels, an X-ray diffractometer was used: a model Seifert 3003 T-T (a cobalt lamp with a characteristic radiation wavelength of λ_cokα_ = 0.17902 nm, Ahrensburg, Germany). Other parameters were:

Supply voltage: Ur = 30÷40 kV,

Current intensity Ir = 30÷40 mA,

Angle range: 2θ = 30÷120°,

Measurement step: 0.1°,

Pulse integration time tr = 10 s.

### 2.5. Surface Geometric Structure and Microhardness Analysis

Surface geometric structure (SGS) and roughness were tested at the Kielce University of Technology and, more specifically, in its Laboratory of Computer Measurements of Geometric Quantities. The optical profiler Talysurf CCI was used for the measurements. It applies the patented algorithm of coherence correlation by Taylor Hobson. Thus, 10 pm axial resolution is attained. The area undergoing testing was 1.65 × 1.65 mm^2^, while the horizontal resolution was 1.65 × 1.65 µm^2^. TalyMap Platinium (version 6.2) software was applied to analyze the surface 3-D images so that the SGS of the layers could be determined. The measurement of SSS microhardness was carried out on a Shimadzu HMV-G Series tester using the Vickers methodology (with a load of 980.7 mN).

### 2.6. Statistical Analysis

From a statistical point of view, the process is described by two explanatory variables, one of which gives the alloying speed and the second of which gives the proportions of the sinter composition. The sinter is a two-component mixture of AISI316L and AISI409L steels, the percentages of which must, in each case, sum up to 100%. For statistical analysis purposes, the share of AISI316L steel was adopted as the related lambda explanatory variable, and thus the share of AISI409L steel is described as (1–lambda). The alloying speed was adopted as the rate explanatory variable. Due to the nature of the available dataset, it is possible to assess the impact of the explanatory variables using a two-way analysis of variance (ANOVA) using a linear model [29,47]. The MINITAB 20.1.3 program was used to analyze and create the related graphs.

## 3. Results and Discussion

### 3.1. Microstructure of Powder, Coating (Cr_3_C_2_–NiCr) and Laser Alloyed Tracks

**Figure 1:**
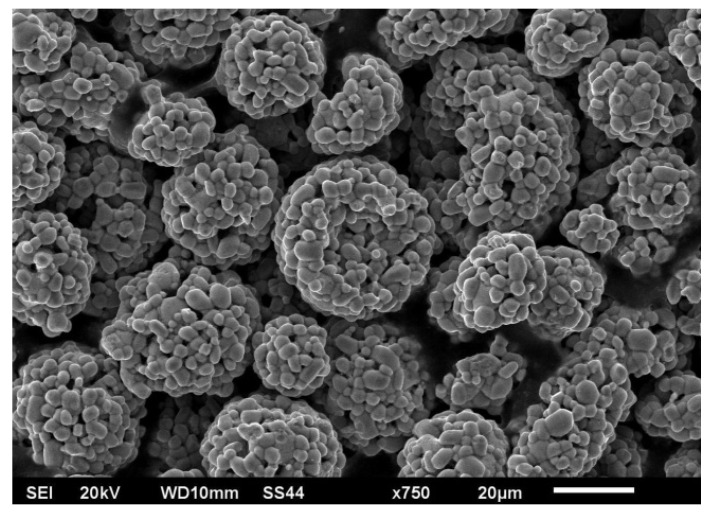
Grains of the Cr_3_C_2_–NiCr powder.

Figure 1 presents the grains of Cr_3_C_2_–NiCr powder obtained by the scanning microscope Jeol JSM–6610LV.

**Figure 2:**
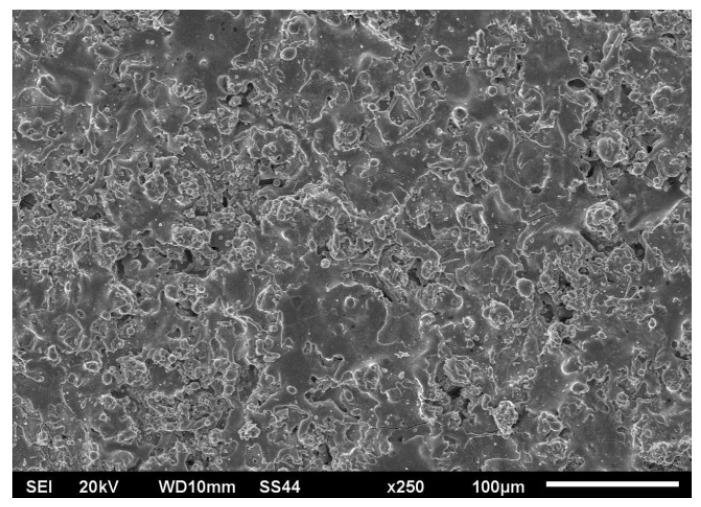
Cr_3_C_2_–NiCr coating surface microstructure (SSS after the APS process).

The surface of the Cr_3_C_2_–NiCr coating (obtained by scanning electron microscopy) before the laser alloying process is shown in Figure 2.

**Figure 3:**
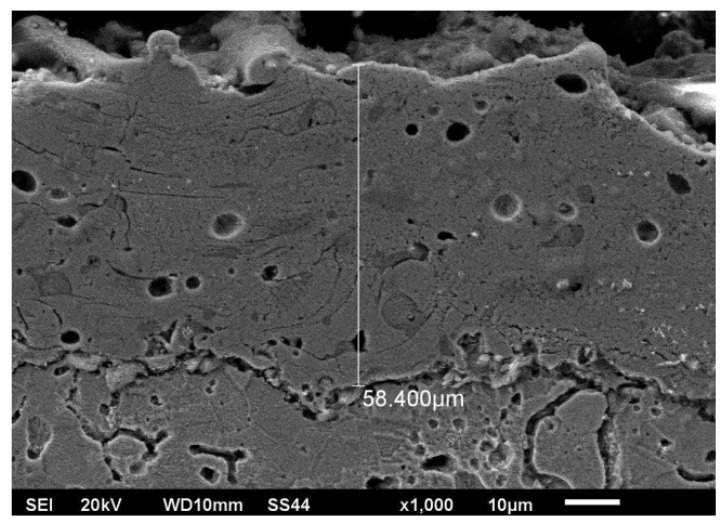
Microstructure of the Cr_3_C_2_–NiCr coating after the APS process for SSS.

Figure 3 shows a cross-section of the microstructure of the Cr_3_C_2_–NiCr coating (the image from Jeol JSM–6610LV scanning microscope). The thickness of the resulting chromium carbide coating was approx. 60 µm.

Structural elements typical of the chosen deposition method (APS) can be observed in the results obtained for this coating: porous lamellar structures with cracks and non-melted particles.

**Figure 4:**
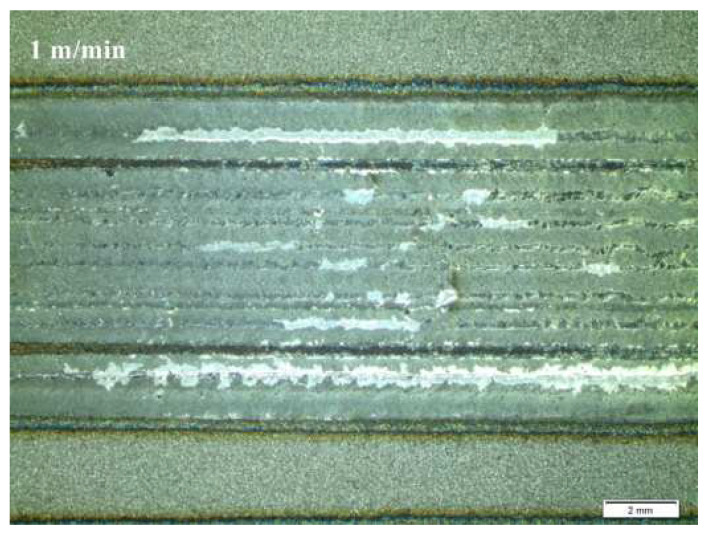
Morphology of the laser-alloyed tracks of surface of SSSs for sample 1.

**Figure 5:**
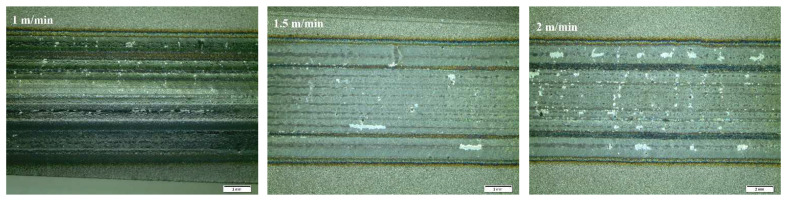
Morphology of the laser-alloyed tracks of surface of SSSs for sample 2.

**Figure 6:**
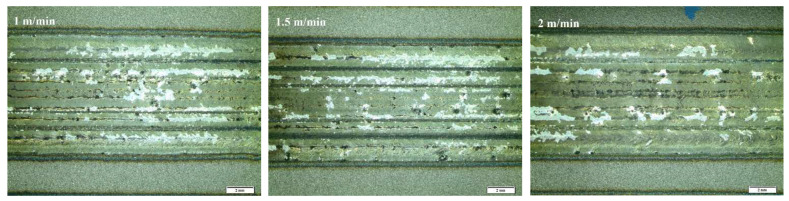
Morphology of the laser-alloyed tracks of surface of SSSs for sample 3.

The impact of laser alloying on the quality of the steel surface was assessed by macroscopic tests. The main criteria for evaluation were the continuity of the band, comparable width, and relatively smooth surface without craters. Figure 4, Figure 5 and Figure 6 present the surface morphology of the laser-alloyed tracks after the laser alloying process for SSSs with Cr_3_C_2_–NiCr coating obtained by an Olympus SZ61 stereo microscope.

**Figure 7:**
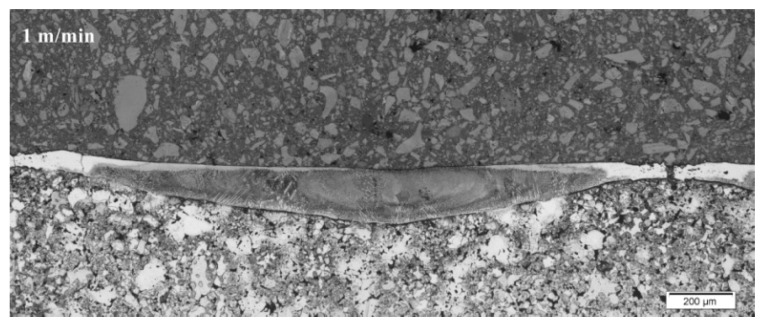
Cross-section of the entire alloyed zone for sample 1.

**Figure 8:**
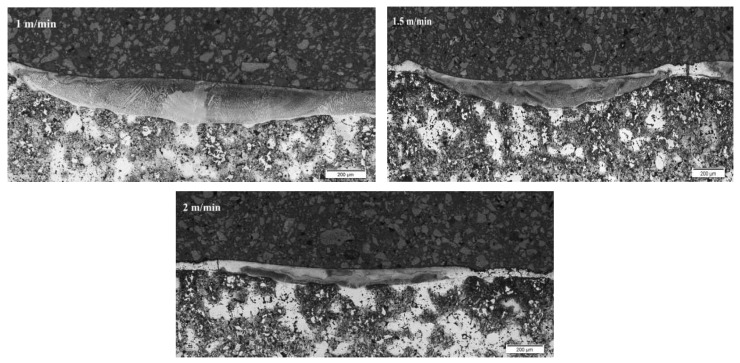
Cross-section of the entire alloyed zone for sample 2.

**Figure 9:**
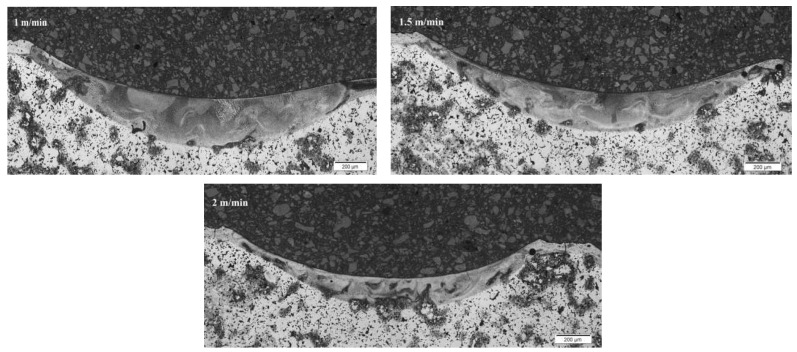
Cross-section of the entire alloyed zone for sample 3.

The metallographic section was prepared, which was etched with copper sulfate solution. Figure 7, Figure 8 and Figure 9 show a fragment of the microstructure of the laser stitch obtained after the laser alloying treatment of the Cr_3_C_2_–NiCr coating (the image was obtained using the Olympus GX41 optical microscope).

In the surface layers, the microstructural examinations of the cross-section showed a homogeneous cellular-dendritic structure. This structure was formed after alloying treatment. Columnar crystals, oriented in the direction of heat transfer, were also observed in the structure. As a result of contact of the alloyed layer with the basic material, a transient zone was created, in which nucleation and an increase in the primary structure crystals occurred.

Table 4 presents the results of the laser alloying process considering the width and depth of the cross-section.

**Figure 10:**
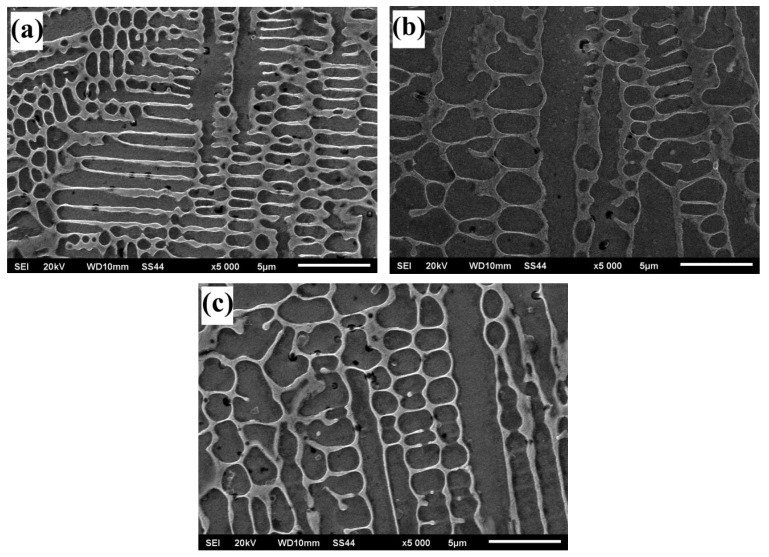
Microstructure of the central part of the alloyed zone for (**a**) sample 1; (**b**) sample 2; (**c**) sample 3.

An example of surface microstructures after the laser alloying process (laser power: 3 kW and speed laser: 1 m/min) for SSS with Cr_3_C_2_–NiCr coating obtained by the Jeol JSM–6610LV scanning microscope is presented in Figure 10.

### 3.2. Chemical and Phase Composition Analysis

Table 5, Table 6 and Table 7 present an analysis of the chemical composition of the surface layers in SSSs with Cr_3_C_2_–NiCr coating after laser alloying. The obtained results made it possible to evaluate three aspects: comparing the surface after treatment, determining the migration of alloying elements during the crystallization process, and determining the chemical composition homogeneity in the surface layers.

**Figure 11:**
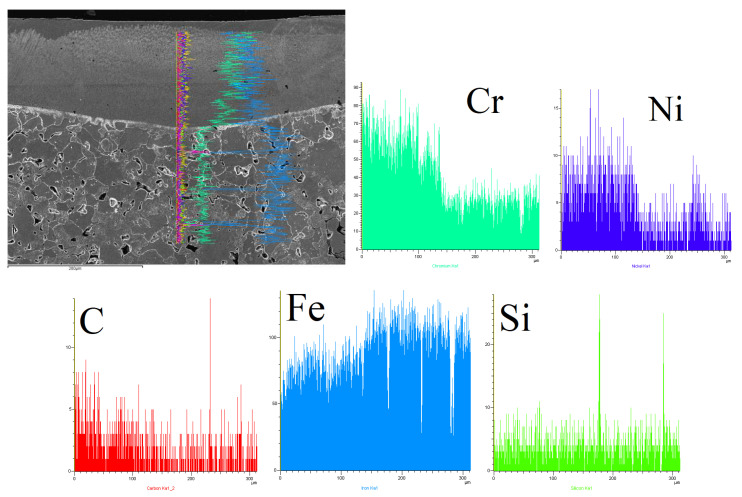
Line mapping analysis EDS on cross-section after laser alloying process for sample 1.

**Figure 12:**
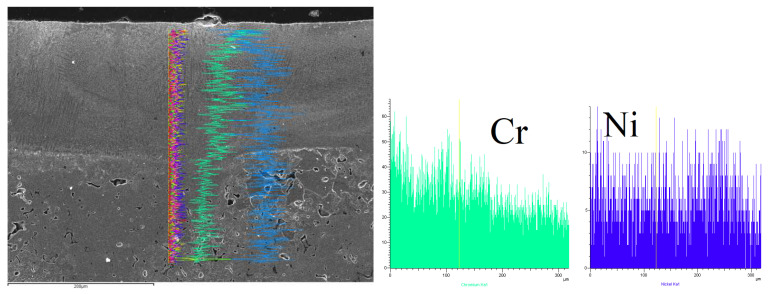

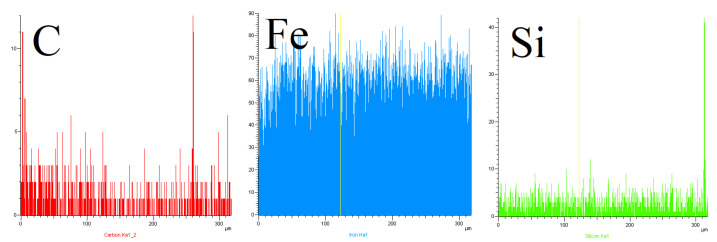
Line mapping analysis EDS on cross-section after laser alloying process for sample 2.

**Figure 13:**
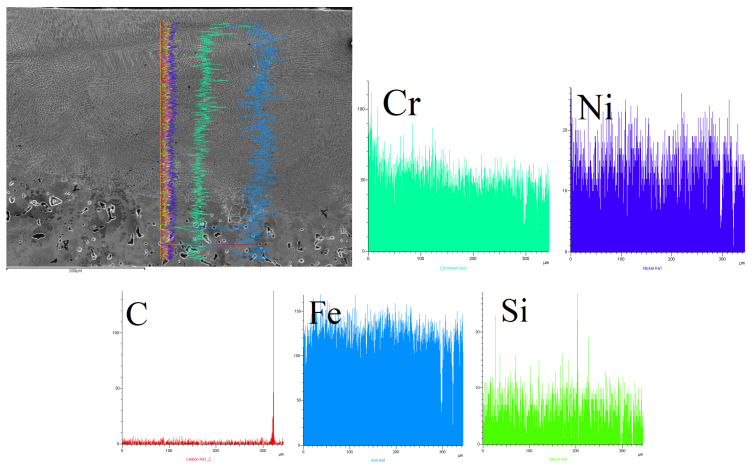
Line mapping analysis EDS on cross-section after laser alloying process for sample 3.

The line mapping analysis in Figure 11, Figure 12 and Figure 13 illustrated the elemental migration across the cross-section after the laser alloying process (laser power: 3 kW and speed laser: 1 m/min) for SSS with Cr_3_C_2_–NiCr coating.

**Figure 14:**
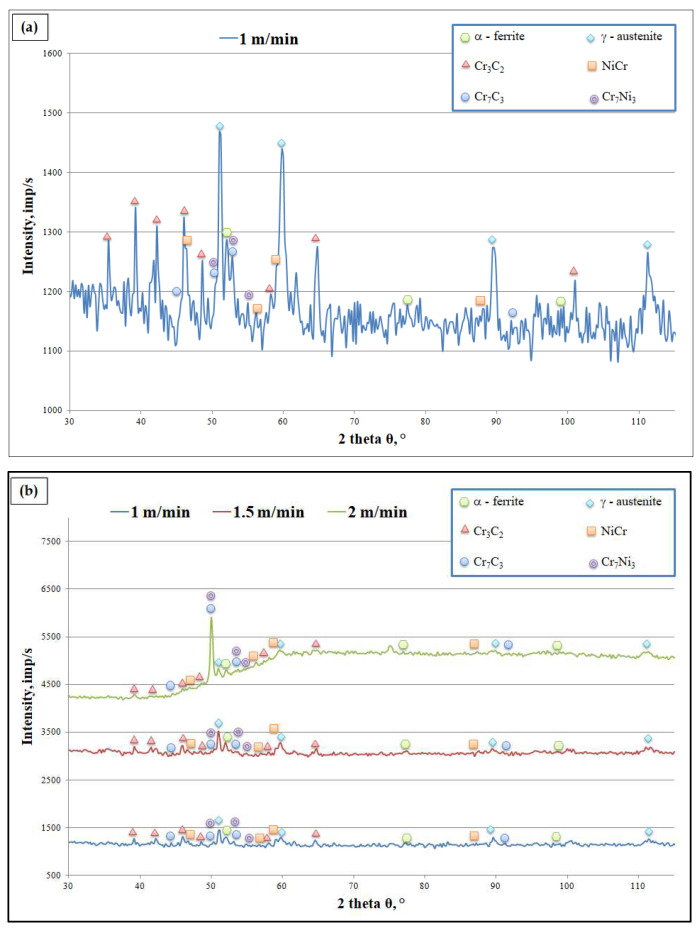

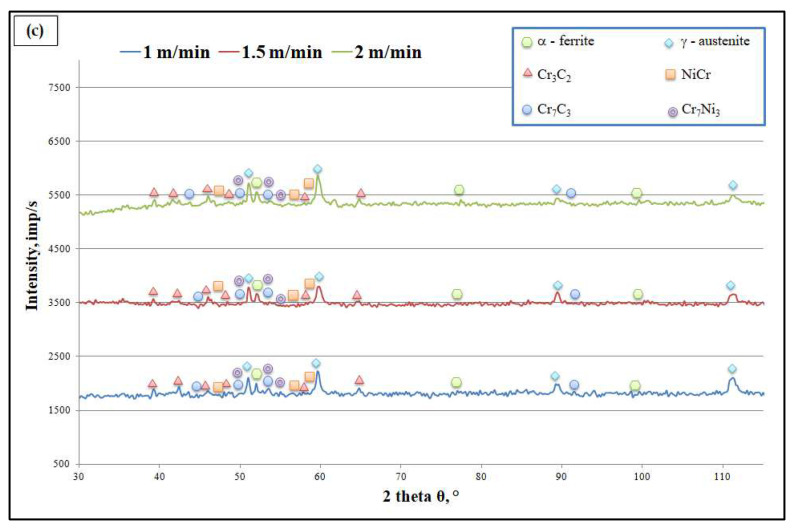
Diffractograms after laser alloying process for (**a**) sample 1, (**b**) sample 2, and (**c**) sample 3.

To determine the phase composition on the laser-alloyed surface layer, an X-ray phase analysis was performed. Results of the identification of the phase composition for samples 1, 2, and 3 are presented in Figure 14. The phase analysis for individual specimens showed the austenitic and ferritic phases with the content proportional to the powders used.

The phase composition analysis for individual specimens revealed the presence of the ferritic phase (cell parameters: a = b = c = 0.286 nm, α = β = γ = 90°) and austenitic phase (cell parameters: a = b = c = 0.359 nm, α = β = γ = 90°), which crystallize in cubic cells. Additionally, analysis revealed presence of the Cr_3_C_2_ phase, which crystallizes in orthorhombic cells (cell parameters: a = 0.55 nm, b = 1.14 nm, c = 0.28 nm, α = β = γ = 90°), and the NiCr phase, which crystallizes in cubic cells (cell parameters: a = b = c = 0.455 nm, α = β = γ = 90°). Phase analysis revealed the Cr_7_C_3_ phase, which crystallizes in orthorhombic cells (cell parameters: a = 0.70 nm, b = 1.21 nm, c = 0.45 nm, α = β = γ = 90°), and the Cr_7_Ni_3_ phase, which crystallizes in tetragonal cells (cell parameters: a = b = 0.87 nm, c = 0.45 nm, α = β = γ = 90°).

### 3.3. Surface Geometric Analysis

**Figure 15:**
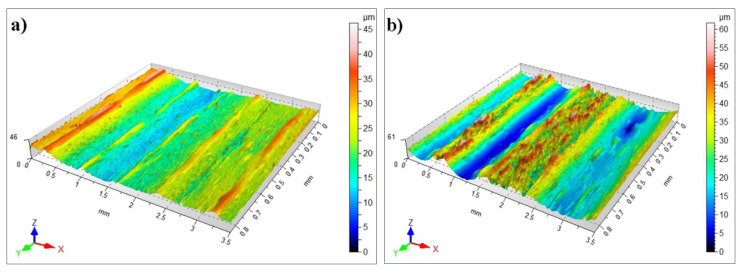

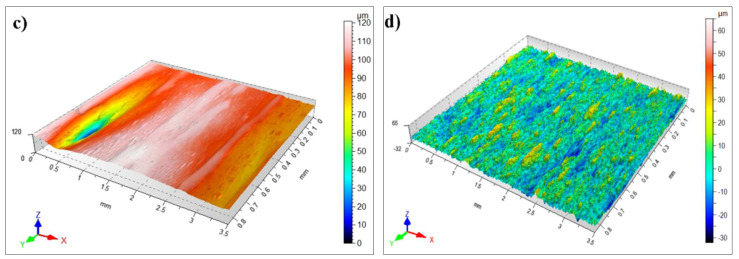
Topography of surface: (**a**) sample 1 (1 m/min); (**b**) sample 2 (2 m/min); (**c**) sample 3 (1 m/min); (**d**) sample of the Cr_3_C_2_–NiCr coating.

The exemplary Figure 15a–c shows the topography images taken on samples that underwent laser alloying, while Figure 15d presents the topography of the Cr_3_C_2_–NiCr surface layer.

Table 8, Table 9, Table 10 and Table 11 summarize the most crucial SGS parameters obtained for the studied specimens.

The sintered stainless steel samples on which the coatings were applied had the value of the Sa parameter in the range of 4.42–17.72 µm. It is a basic amplitude parameter used in the quantitative assessment to determine the considered elements’ physical condition. Treatment by laser led to the reduction of the Sa parameter of the Cr_3_C_2_–NiCr layer after laser alloying (sample 1), which proves that we were dealing with laser smoothing (reduction of the height of the peaks). On the other hand, laser treatment of the Cr_3_C_2_–NiCr layers (samples 2 and 3) elevated the arithmetic mean values of the surface height Sa by 1.5 to 3 times. A tendency of the same nature could be seen in the case of the mean square surface height Sq as well as of the other parameters, such as the maximal values of peak height (Sp), pit height (Sv), and height (Sz).

Supplementary information on the shape of the surface of the tested samples before and after laser treatment can be obtained from the amplitude parameters of skewness coefficient (asymmetry, Sku) as well as concentration coefficient (kurtosis, Ssk). The parameters mentioned above enable the detection of the local elevations and depressions on the surface, as well as any defects (e.g., scratches, delamination). The parameter of the Cr_3_C_2_–NiCr coating after laser alloying (sample 3) had negative values of the Ssk parameter, proving that numerous peaks and depressions are present on the layer. Positive asymmetry Ssk (skewness) values for the coating after laser treatment provide information that we are dealing with a smooth surface devoid of elevations in the shape of a plateau.

The surface inclination values proved to be close to one another. They were all within 2.75÷8.67 for Sku. Thus, the ordinates’ distribution was close to the normal distribution function for all the specimens.

Before the treatment by laser, the Cr_3_C_2_–NiCr coating was characterized by a random isotropic structure (Iz = 92.61%). Laser alloying led to the creation of periodic structures in an area between anisotropic and isotropic structures (Iz = 42.30÷75.69%).

### 3.4. Microhardness

The assessment of mechanical properties was made based on hardness measurements by the Vickers method. Table 12 presents the hardness of the Cr_3_C_2_–NiCr coating, alloying zone, heat-affected zone, and native materials obtained after laser alloying. The results represent the mean of three measurements.

**Figure 16:**
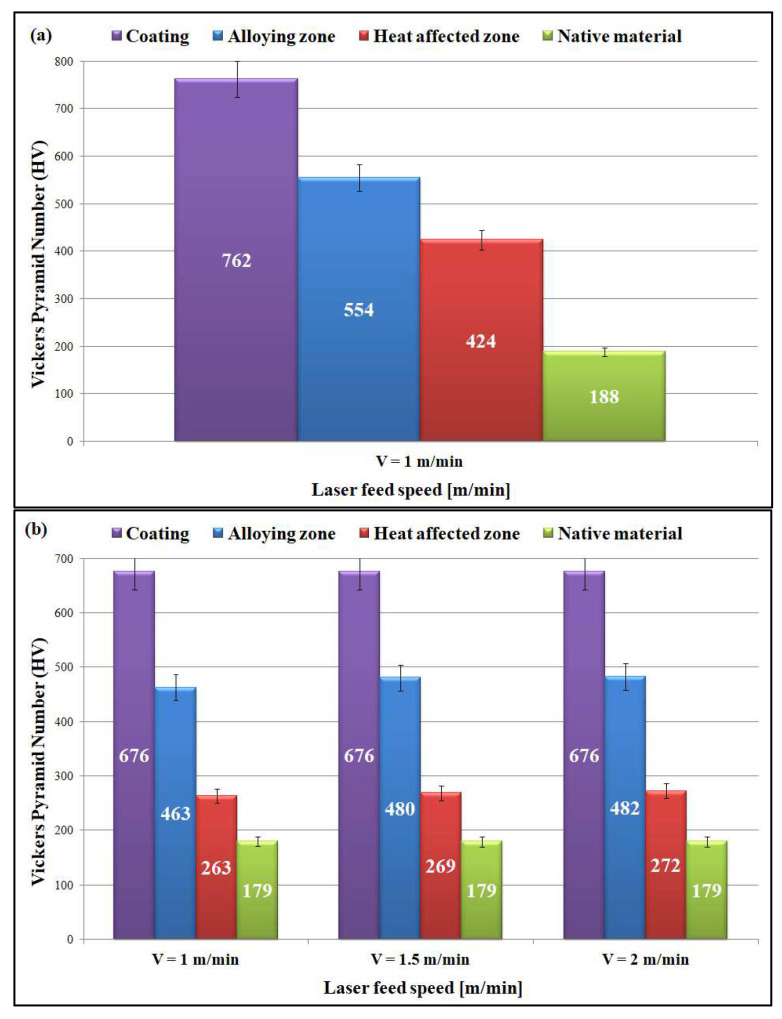

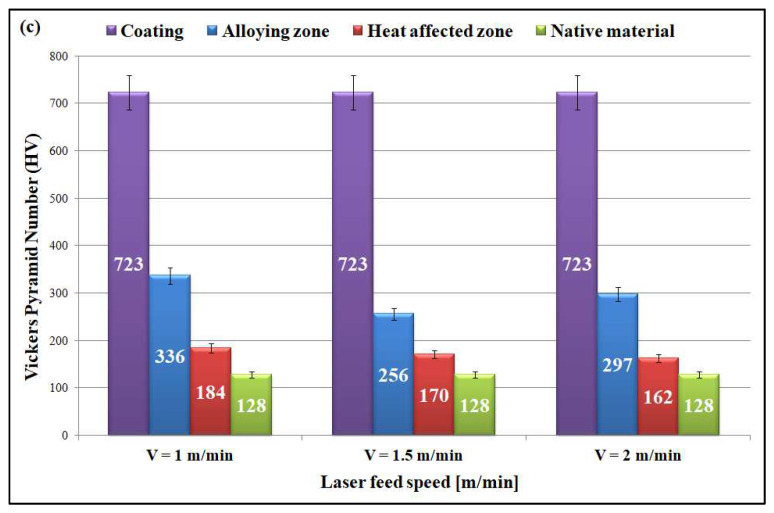
Summary of the results of hardness measurements: (**a**) sample 1; (**b**) sample 2; (**c**) sample 3.

Figure 16 shows a summary of the results of hardness measurements to all samples after laser alloying with Cr_3_C_2_–NiCr coating obtained by the Vickers method.

**Figure 17:**
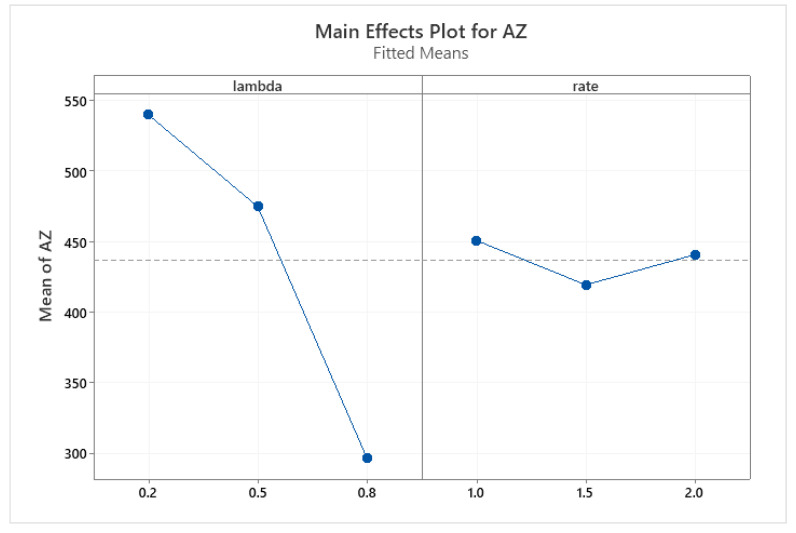
Marginal means plot for AZ hardness with respect to the lambda (share of AISI316L) and rate (speed of alloying) explanatory variables.

**Figure 18:**
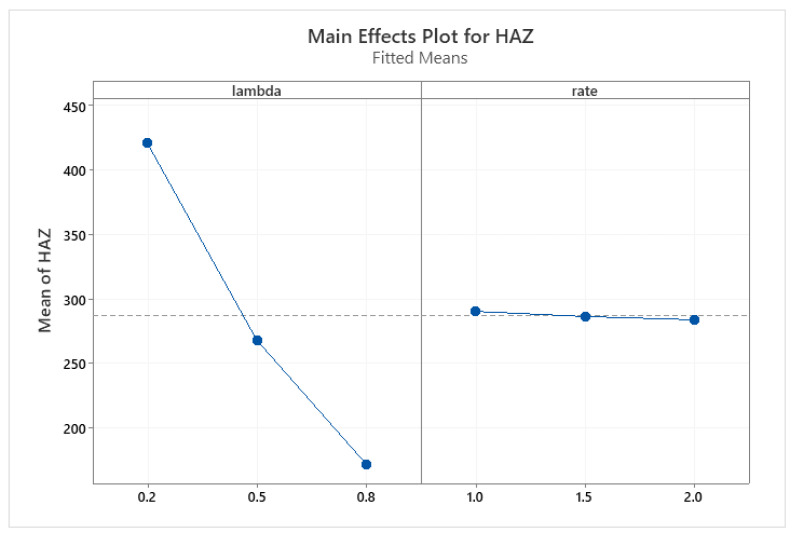
Marginal means plot for HAZ hardness with respect to the lambda (share of AISI316L) and rate (speed of alloying) explanatory variables.

Two-way ANOVA with a linear model for two explanatory variables, lambda (share of AISI316L) and rate (rate of laser alloying process), was applied to the measured hardness of AZ (Table 13) and hardness of HAZ values (Table 14). The plots of the marginal means are provided in Figure 17 (AZ) and Figure 18 (HAZ), respectively.

The analysis clearly showed that the effect of alloying speed is negligible (statistically insignificant), and the composition of the sinter explains the variability of hardness in over 95% (AZ) and over 99% (HAZ). Other uncontrolled factors impact less than 5% (AZ) and less than 1% (HAZ).

The application of the Cr_3_C_2_–NiCr coating and the laser alloying method resulted in the homogenization of the microstructure. This has been confirmed by both microstructural and hardness testing. It has been observed that the preparation of Cr_3_C_2_–NiCr coating on sintered stainless steels led to over 5 times higher hardness and consequently improved the mechanical properties of SSSs. In addition, the use of the laser alloying method improved the properties, as shown by the higher hardness values measured in the alloying zone. The highest hardness values for the alloying zone were obtained for sample 1 (Table 12 and Figure 16a), in which the contribution of the ferritic phase was the highest (20% 316L + 80% 409L).

## 4. Conclusions

It is essential and challenging to select the appropriate coating and associated laser control parameters so that the alloying of the surface layer improves the functional properties of sintered stainless steels. This article presents the strength properties of SSSs and the characteristics of their structure and surface roughness after laser alloying. The research confirms that by applying the Cr_3_C_2_–NiCr coating and modifying the surface layer through the laser alloying method, the mechanical properties of SSSs are improved. The following conclusions were made:

Appropriate laser modification of surface SSSs allows for the homogenization of the structure (homogeneous cellular–dendritic structure), which improves the strength properties (reduction of steel porosity).

The laser treatment of SSSs smoothed the surface of the sintered stainless steel, which is confirmed by the positive values of the surface asymmetry Ssk (skewness) for the coating.

The results obtained in the examination showed that using a Cr_3_C_2_–NiCr coating and laser alloying method causes a hardness increase, resulting in increased wear resistance.

The best hardness values for the alloying zone were obtained for sample 1, in which the contribution of the ferritic phase was the highest (20% 316L + 80% 409L). The laser alloying method used by the authors is a promising proposal for hardening a surface layer of SSSs.

The surface geometric structure parameters of the surface of the Cr_3_C_2_-NiCr coatings after laser alloying are 1.5 to 3 times greater than that of the coatings without this treatment.

The modification proposed in the article allows for broader use of these modern materials.

Further tests should include measurements of internal stresses and tests of resistance to erosion of plasma-sprayed coatings before and after laser alloying.

## Figures and Tables

**Table 1:** Chemical composition of steel powders (% wt.).

Powder Grade	Cr	Ni	Mo	Si	Mn	C	S	Fe
AISI 316L	16.80	12.00	2.00	0.90	0.10	0.022	0.005	Balance
AISI 409L	11.86	0.14	0.02	0.82	0.14	0.020	0.010	Balance

**Table 2:** Percentages of powders in each series of samples (% wt.).

Series of Samples	Powder Grade	
	AISI 316L	AISI 409L
1	20 %	80 %
2	50 %	50 %
3	80%	20 %

**Table 3:** Parameters of laser alloying process.

Parameters	Series of Samples		
	1	2	3
Laser power (kW)	3	3	3
Speed of movement (m/min)	1	1 1.5 2	1 1.5 2
Gas Ar (L/min)	10	10	10
Repetition rate (Hz)	50,000	50,000	50,000
Distance of nozzle to material (mm)	35	35	35
Laser beams (mm)	1 × 10	1 × 10	1 × 10
Operating mode	Continuous	Continuous	Continuous

**Table 4:** Results of the alloyed cross-section.

Series of Samples	Parameters of Laser Alloying Process	Width (μm)	Maximum Depth (μm)
1	1 m/min	9280.00	175.79
2	1 m/min	9690.00	232.69
	1.5 m/min	9540.00	163.01
	2 m/min	9510.00	147.03
3	1 m/min	9800.00	282.55
	1.5 m/min	9520.00	228.21
	2 m/min	9500.00	191.79

**Table 5:** EDX–analysis after laser alloying (AZ—alloying zone, HAZ—heat-affected zone, NM—native material) for sample 1.

Parameters of Laser Alloying Process	Element	Weight (%)		
		AZ	HAZ	NM
1 m/min	Cr	28.46	25.94	12.72
	Ni	6.57	6.20	–
	C	9.67	7.19	5.74
	Fe	53.52	59.13	76.83
	Si	0.47	0.48	2.00

**Table 6:** EDX–analysis after laser alloying (AZ—alloying zone, HAZ—heat-affected zone, NM—native material) for sample 2.

Parameters of Laser Alloying Process	Element	Weight (%)		
		AZ	HAZ	NM
1 m/min	Cr	22.08	20.27	13.51
	Ni	8.30	7.17	5.61
	C	5.87	4.50	2.51
	Fe	61.12	65.46	75.41
	Si	0.51	0.50	0.82
1.5 m/min	Cr	29.98	21.48	12.81
	Ni	8.20	7.32	2.01
	C	11.52	6.71	8.87
	Fe	46.80	60.93	72.59
	Si	0.32	0.71	0.96
2 m/min	Cr	26.97	22.16	14.33
	Ni	8.66	7.66	8.16
	C	10.99	5.82	3.97
	Fe	50.80	61.48	70.04
	Si	0.38	0.72	0.73

**Table 7:** EDX–analysis after laser alloying (AZ—alloying zone, HAZ—heat-affected zone, NM—native material) for sample 3.

Parameters of Laser Alloying Process	Element	Weight (%)		
		AZ	HAZ	NM
1 m/min	Cr	16.85	16.12	15.84
	Ni	9.32	9.88	9.96
	C	1.99	2.46	4.96
	Fe	68.33	68.19	64.84
	Si	0.93	0.70	0.58
1.5 m/min	Cr	27.15	16.35	15.74
	Ni	12.21	10.75	10.01
	C	5.47	4.06	4.09
	Fe	52.08	64.96	65.38
	Si	0.54	1.02	0.99
2 m/min	Cr	16.34	16.36	15.56
	Ni	9.32	9.59	9.81
	C	3.14	2.79	6.57
	Fe	68.24	68.27	64.10
	Si	0.67	0.75	0.70

**Table 8:** Parameters of the SGS of the Cr_3_C_2_–NiCr coating.

SGS Parameters	Cr_3_C_2_–NiCr Coating
Sq (μm)	7.73
Ssk	0.46
Sku	3.56
Sp (μm)	65.10
Sv (μm)	32.07
Sz (μm)	97.19
Sa (μm)	6.01

**Table 9:** Parameters of the SGS after laser alloying (sample 1).

SGS Parameters	Speed of Movement (m/min)
	1
Sq (μm)	5.90
Ssk	0.44
Sku	2.89
Sp (μm)	24.66
Sv (μm)	21.68
Sz (μm)	46.34
Sa (μm)	4.70

**Table 10:** Parameters of the SGS after laser alloying (sample 2).

SGS Parameters	Speed of Movement (m/min)		
	1	1.5	2
Sq (μm)	5.54	11.96	10.07
Ssk	0.68	0.98	0.26
Sku	3.12	4.25	2.75
Sp (μm)	21.21	55.45	36.03
Sv (μm)	14.27	24.26	25.62
Sz (μm)	35.48	79.68	61.65
Sa (μm)	4.42	9.58	8.26

**Table 11:** Parameters of the SGS after laser alloying (sample 3).

SGS Parameters	Speed of Movement (m/min)		
	1	1.5	2
Sq (μm)	13.36	23.27	10.80
Ssk	−1.61	−1.17	−1.07
Sku	8.67	5.32	5.82
Sp (μm)	24.39	41.36	37.86
Sv (μm)	96.56	112.30	76.15
Sz (μm)	120.95	153.67	114.01
Sa (μm)	9.84	17.72	7.85

**Table 12:** Hardness measurements of specimens (AZ—alloying zone, HAZ—heat-affected zone, NM—native material).

Series of Samples	Parameters of Laser Alloying Process	Cr_3_C_2_–NiCr Coating	Hardness (HV 0.1)		
			AZ	HAZ	NM
1	1 m/min	762	554	424	188
2	1 m/min	676	463	263	179
	1.5 m/min		480	269	
	2 m/min		482	272	
3	1 m/min	723	336	184	128
	1.5 m/min		256	170	
	2 m/min		297	162	

**Table 13:** ANOVA of AZ hardness with respect to the lambda and rate explanatory variables.

Source	DF	Seq SS	Contribution	Adj SS	Adj MS	F-Value	*p*-Value
lambda	2	72,171	95.48%	63796	31,898.1	26.78	0.036
rate	2	1036	1.37%	1036	518.2	0.44	0.697
Error	2	2382	3.15%	2382	1191.2		
Total	6	75,589	100.00%				

**Table 14:** ANOVA of HAZ hardness with respect to the lambda and rate explanatory variables.

Source	DF	Seq SS	Contribution	Adj SS	Adj MS	F-Value	*p*-Value
lambda	2	49,494.9	99.42%	40,624.2	20,312.1	164.47	0.006
rate	2	43.0	0.09%	43.0	21.5	0.17	0.852
Error	2	247.0	0.50%	247.0	123.5		
Total	6	49,784.9	100.00%				
